# Systems-level analysis reveals selective regulation of *Aqp2* gene expression by vasopressin

**DOI:** 10.1038/srep34863

**Published:** 2016-10-11

**Authors:** Pablo C. Sandoval, J’Neka S. Claxton, Jae Wook Lee, Fahad Saeed, Jason D. Hoffert, Mark A. Knepper

**Affiliations:** 1 Epithelial Systems Biology Laboratory, NHLBI, National Institutes of Health, Bethesda, MD 20892-1603, USA; 2 National Cancer Center, Goyang Gyeonggi-do, Korea

## Abstract

Vasopressin-mediated regulation of renal water excretion is defective in a variety of water balance disorders in humans. It occurs in part through long-term mechanisms that regulate the abundance of the aquaporin-2 water channel in renal collecting duct cells. Here, we use deep DNA sequencing in mouse collecting duct cells to ask whether vasopressin signaling selectively increases *Aqp2* gene transcription or whether it triggers a broadly targeted transcriptional network. ChIP-Seq quantification of binding sites for RNA polymerase II was combined with RNA-Seq quantification of transcript abundances to identify genes whose transcription is regulated by vasopressin. (View curated dataset at https://helixweb.nih.gov/ESBL/Database/Vasopressin/). The analysis revealed only 35 vasopressin-regulated genes (of 3659) including *Aqp2*. Increases in RNA polymerase II binding and mRNA abundances for *Aqp2* far outstripped corresponding measurements for all other genes, consistent with the conclusion that vasopressin-mediated transcriptional regulation is highly selective for *Aqp2*. Despite the overall selectivity of the net transcriptional response, vasopressin treatment was associated with increased RNA polymerase II binding to the promoter proximal region of a majority of expressed genes, suggesting a nearly global positive regulation of transcriptional initiation with transcriptional pausing. Thus, the overall net selectivity appears to be a result of selective control of transcriptional elongation.

In mammals, the peptide hormone vasopressin controls renal water excretion, largely through its actions in the renal collecting duct to regulate the molecular water channel aquaporin-2. Two modes of physiological regulation have been identified: 1) short-term regulation by membrane trafficking^1^,^2^ and 2) long-term regulation involving vasopressin-induced changes in the abundance of the aquaporin-2 protein^3^,^4^,^5^. Defects in the long-term regulation have been implicated in multiple water balance disorders^3^. Therefore, the long-term regulatory action of vasopressin is of prime importance to understand disease mechanisms. Here, we use the methods of *systems biology*, specifically *next-generation DNA sequencing* (both RNA-seq and ChiP-seq for RNA polymerase II) combined with computational analysis, to address whether the regulation of aquaporin-2 expression in collecting duct cells (i.e. mouse mpkCCD cells) by vasopressin is due chiefly to transcriptional control and whether the regulatory process is selective for aquaporin-2. The basic idea of systems biology is to investigate a biological process by studying all relevant components together in parallel to discover mechanism^6^. Thus, using deep sequencing techniques, we can get key information about *Aqp2* gene regulation in the context of data regarding every other expressed gene.

*Next-generation DNA sequencing* is a recently developed methodology that enables relatively inexpensive large-scale DNA sequencing, and is practical for individual small laboratories pursuing targeted questions like the one in this paper^7^,^8^. *RNA-seq* is an approach, based on deep sequencing of DNA, that allows complete transcriptomes to be identified for a given cell type and permits quantitative analysis of experimental effects on every transcript. *ChIP-seq* is an approach that comprehensively identifies DNA binding sites for particular proteins over the entire genome. It combines the method of chromatin immuno-precipitation using antibodies specific to a particular protein (here, the large subunit of RNA polymerase II, Polr2a) with deep sequencing.

Cultured mpkCCD cells have been a useful model for understanding regulatory processes in principal cells of the mammalian collecting duct^9^ and show large increases in aquaporin-2 mRNA and protein following long-term exposure to vasopressin similar to native collecting duct cells^4^,^10^. Thus, mpkCCD cells provide a suitable model to investigate the mechanisms whereby vasopressin increases aquaporin-2 protein abundance in the renal collecting duct.

Prior studies have demonstrated that vasopressin increases the steady-state half-life of the aquaporin-2 protein^11^,^12^, but the increase in half-life from 9 to 14 hours is not sufficient to explain the 10-fold or more increase in aquaporin-2 protein normally seen in response to vasopressin^11^. Vasopressin also increases the translation rate of aquaporin-2^11^, but the increase appears to be due chiefly to an increase in aquaporin-2 mRNA levels rather than translational control *per se*. Although vasopressin increases aquaporin-2 mRNA levels^4^,^13^,^14^,^15^, it has not been established with certainty whether the increase is due to increased transcription of the *Aqp2* gene or is due to a decrease in the degradation rate of the aquaporin-2 mRNA. Data from prior studies in cultured mpkCCD cells suggest that vasopressin does not alter aquaporin-2 mRNA stability^16^,^17^, implicating transcriptional regulation by the process of elimination. If true, then we would expect that RNA polymerase II, the polymerase responsible for production of mRNA, would manifest increased DNA binding to the gene body of the *Aqp2* gene in response to vasopressin. To address this, we used ChIP-seq to identify and quantify RNA polymerase II binding throughout the genome. The comprehensive nature of this method provides information about the selectivity of vasopressin’s effect on *Aqp2* gene transcription. To provide additional data on the transcriptional effects of vasopressin signaling in collecting duct cells using an independent methodology, we also carried out RNA-seq to identify and measure transcriptome-wide mRNA abundance changes in response to vasopressin.

Overall, the results show a highly significant increase in RNA polymerase II occupancy across the *Aqp2* gene body associated with a large increase in aquaporin-2 mRNA. Of only 35 genes with coincident changes in RNA polymerase II binding and mRNA levels in response to vasopressin, the increases for *Aqp2* were by far the greatest, indicating a highly selective effect of vasopressin signaling on *Aqp2* gene transcription. Interpreting the results in terms of Shannon information content^18^, the greater selectivity would require more transcription factor binding sites than would be necessary for non-selective, i.e. more widespread, regulation. Although the major focus of this paper is the regulation of the Aqp2 gene, an important by-product of the work is a comprehensive listing of mRNA abundances and RNA polymerase II occupancy for genes expressed in cultured mpkCCD cells. This information is provided to users via a publically accessible web page.

## Results

### Confirmation of vasopressin response

**Figure 1:**
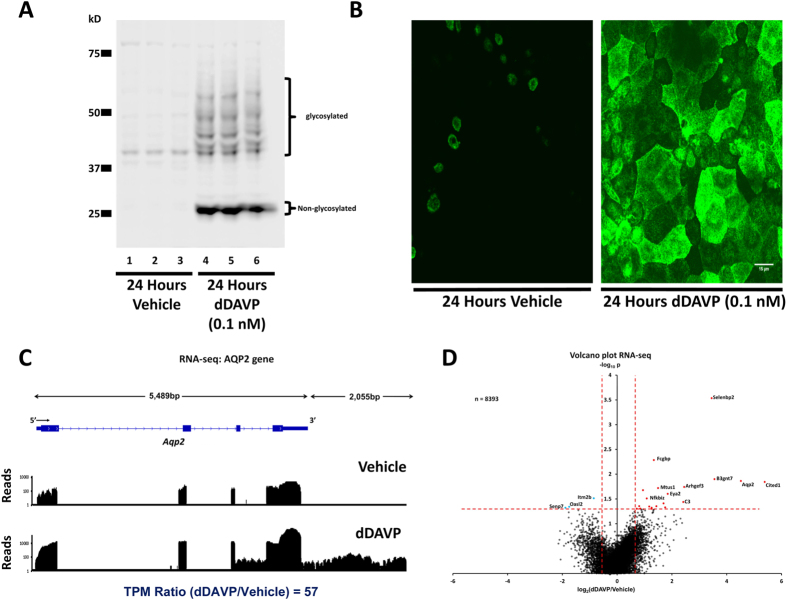
The V2-receptor-specific vasopressin analog dDAVP (0.1nM for 24 hours) increases the abundance of aquaporin-2 (AQP2) protein and mRNA in mouse collecting duct cells (mpkCCD). (**A**) Western blot shows large increase in AQP2 protein (Vehicle, Lanes 1, 2 and 3; dDAVP, Lanes 4, 5 and 6). (**B**) Confocal images of immunofluorescence immunocytochemistry for AQP2 confirms the large increase in AQP2 protein abundance in response to dDAVP. Scale, 15 μm. (**C**) RNA-seq data for Aqp2 gene shows large increase in AQP2 mRNA. RNA-seq reads for a single pair of samples are mapped to exon-intron structure of gene for vehicle-treated cells (above) and dDAVP-treated cells (below). Most reads are mapped to 3′-most exon because reverse transcripton was primed with oligo-dT. (Supplemental Fig. 1 shows the same mapping on a linear scale.). (**D**) Volcano plot for RNA-seq data for all 8393 detectable transcripts shows that a relatively small fraction of transcripts are regulated by vasopressin, but that AQP2 mRNA is increased by a relatively large amount. The horizontal axis shows the mean log_2_(dDAVP/Vehicle) for 9 pairs of samples. The vertical axis shows −log_10_P for t-tests for each gene. Vertical dashed lines show 95% confidence interval for random variation based on Vehicle:Vehicle comparisons (2 × SD). The use of two selection factors represented on x- and y- axes provides stringent identification of vasopressin-responsive transcripts in upper right (red points) and upper left (blue points).

These studies were done in cultured mouse collecting duct cells (mpkCCD) re-cloned in our laboratory to maximize the response to vasopressin^10^. Figure 1 shows preliminary experiments using immunoblotting (Fig. 1A) and immunofluorescence immunocytochemistry (Fig. 1B) confirming that these cells respond to the V2 receptor-selective vasopressin analog dDAVP (0.1 nM) with a large increase in aquaporin-2 protein abundance after 24-hr exposure.

### Profiling of mRNA abundance changes in response to vasopressin using RNA-seq

To quantify changes in aquaporin-2 mRNA in response to dDAVP and compare the responses to those of all other genes, we used RNA-seq. Nine replicates were analyzed in both dDAVP- and vehicle-treated cells to maximize the ability to detect small changes. Figure 1C shows the mapping of RNA-seq reads to the aquaporin-2 gene (*Aqp2*) for one pair of samples, revealing a marked increase with dDAVP. This figure uses a logarithmic scale for the vertical axis to view the pattern of mapped reads both in the presence and absence of dDAVP. **(A** linear version of this figure is provided as Supplementary Fig. 1). The reads mapped to all four exons, although most mapped to the final exon because the reverse transcription step used oligo-dT as a primer. When the reads mapped to *Aqp2* are normalized by the total number of reads for all genes, the log_2_(dDAVP/Vehicle) value for all replicates was 4.52 ± 0.85 (approximately 22-fold increase, n = 9), consistent with the prior conclusion that vasopressin markedly increases aquaporin-2 mRNA levels in mpkCCD cells^4^,^19^. Bedgraph files are provided as Supplementary Data Sets to allow readers to view the mappings of RNA-seq reads to other genes. All supplementary data may be downloaded at https://helixweb.nih.gov/ESBL/Database/AVP_Transcr/.

Figure 1D shows a “volcano plot” indicating changes in normalized mRNA abundance levels (TPM values) for all genes. The horizontal axis shows the log_2_(dDAVP/vehicle) values for all transcripts. The vertical axis of the plot shows P values from t-tests comparing all dDAVP-treated samples to all vehicle-treated samples. The dashed red lines indicate threshold values (P < 0.05 for vertical axis and log_2_(dDAVP/vehicle) > 2 × SE^cc^ for horizontal axis, where SE^cc^ is the median standard deviation for 3 separate control:control pairs). For this study, both thresholds must be exceeded to consider the transcript significantly changed in abundance, providing a relatively stringent criterion favoring false negatives over false positives. Supplementary Table 1 lists mRNA abundances (TPM values) for all 8393 expressed genes. Note that more transcripts show increases than decreases in response to dDAVP, consistent with prior findings using Affymetrix microarrays^19^.

### Profiling of RNA polymerase II binding across the genome using ChIP-Seq

**Figure 2:**
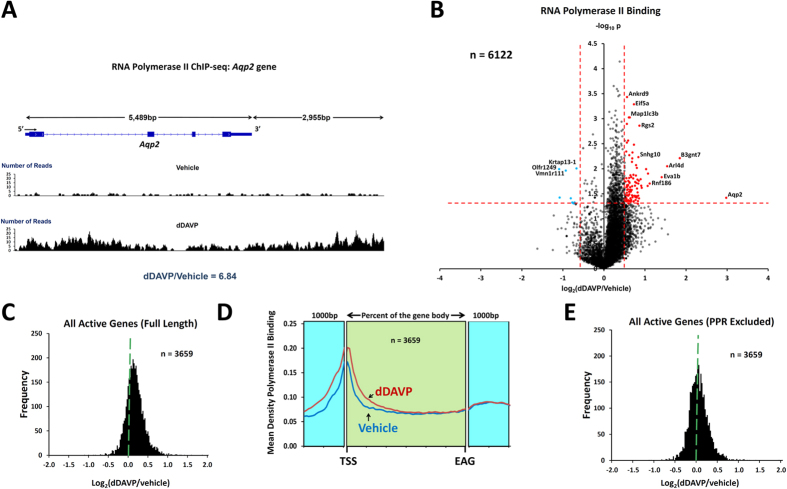
RNA Polymerase II ChIP-seq. (**A**) Mapped reads for typical RNA Polymerase II ChIP-seq samples show increased Polr2a binding to *Aqp2* gene. Reads for a single pair of samples are mapped to exon-intron structure of gene for vehicle-treated cells (above) and dDAVP-treated cells (below). In this example, the dDAVP:Vehicle ratio is 6.8 (unnormalized reads mapped to gene body). (**B**) Volcano plot for RNA Polymerase II ChIP-seq data for top 6122 genes shows marked asymmetry. More genes show increased RNA Polymerase II binding with dDAVP than show decreased binding. The horizontal axis shows the mean log_2_(dDAVP/Vehicle) for 3 pairs of samples. The vertical axis shows –log_10_P for t-tests for each gene. Vertical dashed lines show 95% confidence interval for random variation based on Vehicle:Vehicle comparisons (2 × SD). The use of two selection factors represented on x- and y- axes provides stringent identification of vasopressin-responsive transcripts in upper right (red points) and upper left (blue points). (**C**) Histogram for dDAVP:Vehicle ratio for RNA Polymerase II binding shows a median value >0 (dashed green line). Values were from the 3659 genes with successful measurements in all RNA-seq and ChIP-seq samples. (**D**) Composite tracings for dDAVP and Vehicle showing average RNA Polymerase II read densities over all gene bodies from TSS (transcription start site) to EAG (end of annotated gene). Difference in Polr2a binding is apparent only at 5′-end. Data are also plotted for 1000 bp upstream and 1000 bp downstream from gene body for comparison. (**E**) Histogram for dDAVP:Vehicle ratio for RNA Polymerase II binding to gene bodies beyond the +400 position (relative to TSS) shows a median value ~0 (dashed green line).

To quantify changes in RNA polymerase II binding to each annotated gene in response to dDAVP and compare the *Aqp2* responses to those of all other genes, we used ChIP-seq. This method maps the polymerase as it traverses the gene from 5′ to 3′ to produce mRNA. Thus, changes in transcription can be expected to be associated with coordinate changes in RNA polymerase II binding. The cells are harvested after exposure to dDAVP (0.1 nM) or its vehicle for 24 hours. We profiled 3 experimental pairs (vehicle vs dDAVP). Figure 2A shows the mapping of RNA polymerase II ChIP-seq reads to the *Aqp2* gene for one vehicle:dDAVP experimental pair done at the same time as the RNA-seq measurements shown in Fig. 1C. Exposure of the cells to dDAVP can be seen to have increased RNA polymerase II binding to the entire gene from the promoter just upstream of the gene body, through the gene body and into the downstream 3′ region. Using only reads that map to the gene body, there was a 6.8-fold increase in RNA polymerase II binding in this experiment. The value for log_2_(dDAVP/vehicle) over all three experiments was 2.78 ± 0.46 (P < 0.05). This result therefore supports the conclusion that the vasopressin-induced increase in aquaporin-2 mRNA abundance is, at least in part, due to increased transcription.

**Figure 3:**
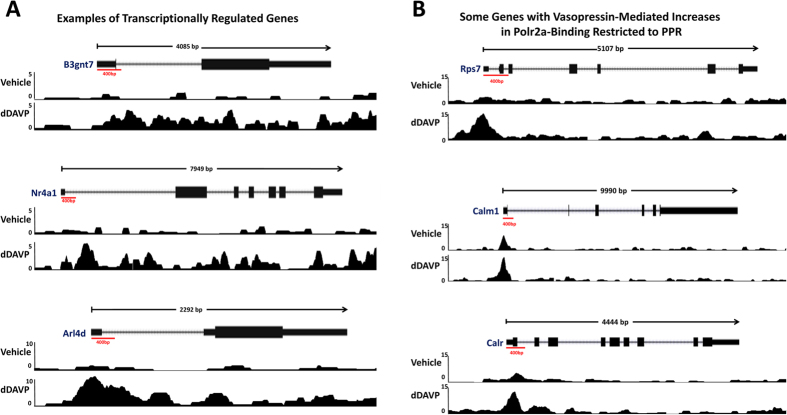
Examples of Polr2a binding to gene bodies of selected expressed genes. (**A**) Three genes for which Polr2a binding is increased along entire gene body: *B3gnt7*, top; *Nr4a1*, middle; *Arl4d*, bottom. All three exhibited concomitant increases in mRNA abundances. (**B**) Three genes for which Polr2a binding is increased only in promotor proximal region: *Rps7*, top; *Calm3*, middle; *Calr*, bottom. None exhibited concomitant increases in mRNA abundances.

Figure 2B shows a volcano plot for dDAVP-induced changes in RNA polymerase II binding to individual gene bodies (n = 6122 genes). The chief observation is the widespread, almost global, increase in RNA polymerase II binding in response to dDAVP, producing a strong asymmetry. (The full data set is available in Supplementary Table 2). The shift is further documented in a histogram of log_2_(dDAVP/vehicle) values (Fig. 2C). The extreme asymmetry is surprising because the mRNA expression results (previous Affymetrix expression arrays^19^ and the RNA-seq data in this study) show a much more subtle asymmetry and relatively few mRNA levels significantly increased in response to vasopressin. An explanation for this apparent discrepancy is provided when the general RNA polymerase II binding pattern from 5′ to 3′ is examined (Fig. 2D). This figure plots the average RNA polymerase II binding over all genes quantified. As shown, RNA polymerase II binding is dominant near the 5′ end of genes (near the transcription start site [TSS]) and the increase in RNA polymerase II binding in response to dDAVP (when averaged over all genes) is limited to this region, the so-called *promotor-proximal region* (PPR). This finding suggests that, in most genes, vasopressin-induced increases in RNA polymerase II binding at the PPR is not associated with increased production of full length transcripts. Figure 2E shows a histogram of log_2_(dDAVP/vehicle) values for RNA polymerase II binding *beyond* the PPR (i.e. reads beyond the first 400 bp of the gene bodies) for all genes. As seen, the strong rightward shift was eliminated, leaving relatively few genes that show changes in response to vasopressin. Some examples of the RNA polymerase II binding profiles for selected genes are shown in Fig. 3A (transcriptionally regulated) and Fig. 3B (increased binding in response to dDAVP only in the PPR). All other profiles can be found at https://helixweb.nih.gov/ESBL/Database/Vasopressin/ (click on official gene symbol).

**Figure 4:**
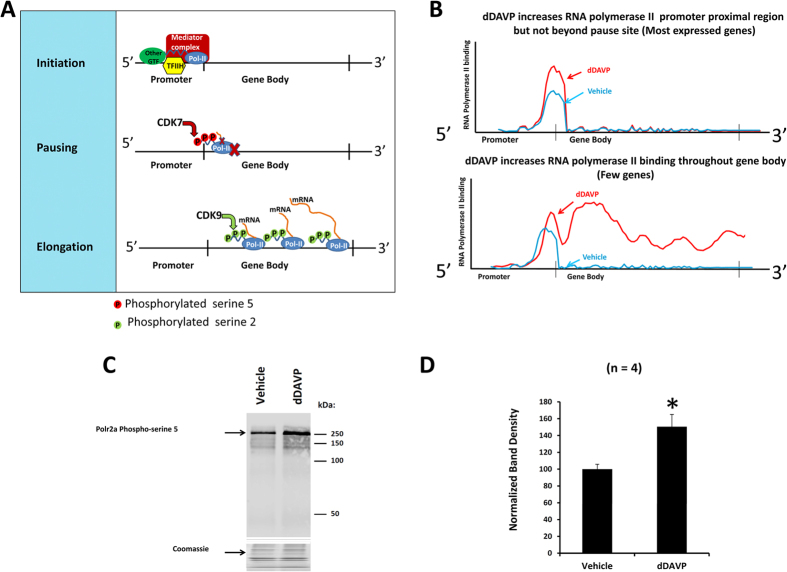
Cartoons showing simplified views of transcriptional regulation and the effects of vasopressin on transcription in mpkCCD cells. (**A**) Three elements of transcription are initiation (top), pausing (middle) and elongation (bottom). Initiation involves localization of the RNA polymerase II complex (Pol-II) at the TSS guided in part by General Transcription Factors (GTFs) including TFIIH and the Mediator Complex. At initiation, Polr2a is phosphorylated at Serine-5 positions in the 52 heptad repeats making up the COOH-terminal domain by cyclin-dependent kinase 9 (CDK9). Most genes exhibit pausing of Pol-II in the promoter proximal region indicated by the red X. Pause release occurs in part because of Polr2a phosphorylation at Serine-2 positions of the COOH-terminal heptads by CDK7, promoting elongation. During elongation, Polr2a-specific phosphatases dephosphorylate at Serine-5 positions. (**B**) The two types of vasopressin-mediated regulation exhibited in this study. In most expressed genes (top), vasopressin increased RNA polymerase II binding only in the promoter proximal region. This pattern and the increase in Ser5 phosphorylation in Polr2a support the conclusion that vasopressin signaling triggers a broad increase in transcriptional initiation for most genes. For a few genes (bottom), vasopressin increased RNA polymerase II binding throughout the gene body pointing to highly selective regulation of elongation. (**C**) Immunoblot for Polr2a phosphorylated at Ser5 positions in COOH-terminal domain shows increase with vasopressin. Coomassie-stained gel shows identical input protein. (**D**) Mean band density for pSer5-Polr2a was significantly increased over 4 replicates. *P < 0.05, t-test.

The observations shown in Figs 3 and 4 can be understood on the basis of current knowledge about mechanisms involved in transcriptional regulation^20^, summarized in Fig. 4A. Fundamentally, regulation of transcription can occur by two distinct mechanisms, viz. regulation of transcriptional initiation and transcriptional elongation^20^. Transcriptional initiation occurs upon the Mediator-dependent binding of RNA polymerase II along with general transcription factors to the promoter region of the gene to form the so-called preinitiation complex, which begins the transcription process. The latter is the consequence of transcriptional pausing and its release^21^. Pausing is the result of either transient or sustained halting of RNA polymerase II in the promotor proximal region (PPR), i.e. near the 5′-end of the gene body. Pause release allows RNA polymerase II to progress beyond the PPR to produce a full transcript. The observations in the present study are summarized in Fig. 4B. We have found that most expressed genes show increases in RNA polymerase II binding at the PPR in response to dDAVP as exemplified in the top portion of Fig. 4B. This finding is consistent with an effect of vasopressin to accelerate transcriptional initiation globally. However, only a few genes (including *Aqp2*, *B3gnt7*, *Arl4d* and *Nr4a1*) show increased RNA polymerase II binding beyond the PPR in response to dDAVP (Fig. 4B, bottom), suggesting that acceleration of transcriptional elongation by vasopressin is very selective.

Transcriptional initiation is associated with Cdk7-dependent phosphorylation of the large subunit of RNA Polymerase II (Polr2a) at multiple serines corresponding to position 5 in the 52 heptad repeats at the COOH-terminal tail of Polr2a^22^ (Fig. 4A). Cdk7, as a subunit of the General Transcription Factor IIH complex, is a component of the transcriptional initiation complex. Later, as elongation proceeds, Ser5 phosphorylation is reversed by phosphatases. Thus, if transcriptional initiation is increased by vasopressin as implied by the data in Fig. 2D, then we would expect an increase in Ser5 phosphorylation in response to dDAVP. Figure 4C shows an immunoblot using a phosphospecific antibody to Ser5 with and without dDAVP. (The material loaded on the gels was the output from the Polr2a ChIP procedure carried out as for the ChIP-seq.) The band density for the Ser5-phosphorylated form of Polr2a was significantly increased by dDAVP exposure (Fig. 4D), consistent with a broad increase in transcriptional initiation with extensive pausing.

### Integration of RNA-seq and ChIP-seq responses to vasopressin

**Figure 5:**
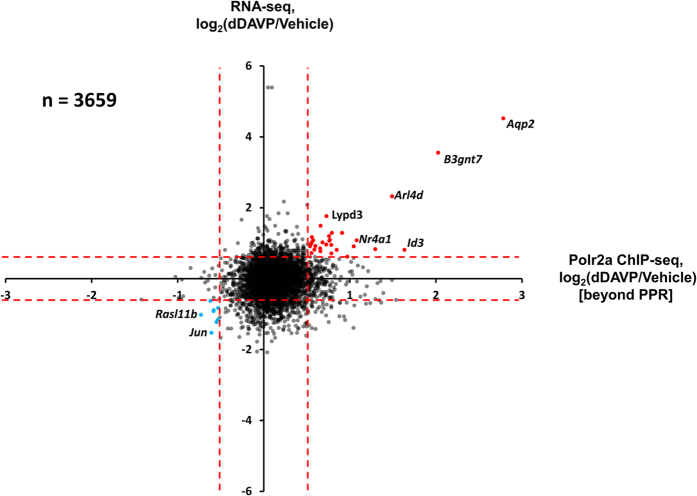
Scatterplot, showing log_2_(dDAVP:Vehicle) values for RNA-seq versus log_2_(dDAVP:Vehicle) values for RNA Polymerase II binding to gene body beyond the promoter proximal region (PPR) shows net transcriptional regulation for only a few genes including *Aqp2*. Dashed red lines indicate 95% confidence for Vehicle:Vehicle comparisons as indicated in Figs 2D and 3B. Red points indicate upregulation by vasopressin. Blue points indicate downregulation. All regulated genes are listed in Tables 1 and 2.

To provide a stringently vetted listing of genes undergoing net regulation of transcription in response to dDAVP, we compared the RNA-seq and ChIP-seq responses (Fig. 5). The genes in the right upper region (significantly increased in both measures, n = 29) are reported in Table 1 and those in the lower left region (significantly decreased in both measures, n = 6) are reported in Table 2. Interestingly, Fig. 5 shows that aquaporin-2 was the maximum responder for both measures. In fact, the magnitudes of the aquaporin-2 responses greatly exceeded the changes seen in every other gene, suggesting that the transcriptional regulatory network for vasopressin is highly selective for the *Aqp2* gene. A summary of all data is provided as a publically accessible webpage at https://helixweb.nih.gov/ESBL/Database/Vasopressin/. The data have also been deposited in *Gene Expression Omnibus* (GEO) (http://www.ncbi.nlm.nih.gov/geo/query/acc.cgi?acc=GSE79584). (Curated data are available at https://helixweb.nih.gov/ESBL/Database/AVP_Transcr/.)

Note that in Fig. 5 there are genes whose transcript abundances are increased or decreased without significant changes in RNA polymerase II binding downstream from the PPR (Supplementary Table 3), consistent with previous observations demonstrating that there is extensive post-transcriptional regulation in the overall dDAVP response^19^. Conceivably, some of these mRNA abundance changes could be due to changes in mRNA stability. One predictor of control via mRNA stability changes is the presence of AU-rich elements (ARE) in the 3′-untranslated region^23^. Using software called ARED (URL: http://brp.kfshrc.edu.sa/AredOrg/), we found putative AREs in 21 of 149 (14.1%) upregulated transcripts from Fig. 5, compared with 226 of 2677 transcripts (8.4%) that were not upregulated (significantly increased by Fisher Exact Test, P = 0.024; analysis in Supplementary Table 4). No ARE was found in AQP2 mRNA, consistent with the view that the stability of the aquaporin-2 transcript is not regulated in response to vasopressin^16^,^17^.

Vasopressin-mediated effects on transcript abundances in collecting duct cells were previously investigated using SAGE analysis^24^, cDNA arrays^25^ and Affymetrix expression arrays^19^. Comparison of the Affymetrix data for mpkCCD cells^19^ with the values in Tables 1 and 2 (RNA-seq data) shows a high degree of correlation between the two techniques. 21 of the vasopressin-responsive transcripts in Tables 1 and 2 (RNA-seq data) were previously found to be significantly changed (in the same direction) in the Affymetrix study: *Aqp2, Arl4d, Atp1b1, B3gnt7, Bhlhe40, Cdk18, Gadd45b, H6pd, Id3, Jun, Krt19, Lypd3, Nfkbiz, Nr4a1, Rasl11b, Sat1, Selm, Sik1, Sptbn2, Srxn1*, and *Tspan1*. The comparison shows that, in general, the dDAVP:vehicle ratios were greater with RNA-seq analysis, suggesting that there is a significant degree of ratio compression with the microarrays (Supplementary Table 5). The SAGE study^24^ identified a limited number of vasopressin-regulated transcripts in mpkCCD cells many of which were found to be regulated in the same direction in our study including *Hilpda, Avpi1*, and *Tsc22d3* (GILZ). In a cDNA array study in which whole inner medullary transcripts were measured in mice^25^, 38 transcripts underwent changes in response to vasopressin that were in line with those seen in the present study including *Vamp8, Zfp36l1*, and *Tsc22d4*. Zpf36l1 is a member of a family of proteins that binds to AU-rich regions (AREs) at the 3′-ends of transcripts and regulates their stabilities^26^.

### Classification of vasopressin-regulated genes

The regulated genes listed in Tables 1 and 2 group into functional categories summarized in Supplemental Table 6. There were four transcription factors that were increased, viz., *Bhlhe40, Id3, Nr4a1*, and *Zfp750*, as well as one that was decreased, viz. *Jun*. All of these have potential roles in regulation of *Aqp2* gene transcription. There are two protein kinases, viz., *Cdk18* and *Sik1*, which have potential roles in vasopressin signaling. There are two small GTP-binding protein transcripts that were found to be regulated by vasopressin: *Arl4d (*increased) and *Rasl11b (*decreased) with potential roles in vesicle trafficking and regulation of the actin cytoskeleton. Consistent with the role of vasopressin in regulation of apoptosis in collecting duct cells^27^, transcripts for several genes involved in regulation of apoptosis were increased (*Nfkbiz, Osgin1, Sik1*, and *Tob1*) or decreased (*Gadd45b*) by vasopressin. Tables 1 and 2 also include several long non-coding RNAs (lncRNAs) that themselves can play roles in transcriptional regulation^28^. Contrary to their name, many lncRNAs code for short regulatory peptides (small open reading frames or smORFs) that can modulate the activity of ion transporters in cells^29^. Putative smORFs coded by the five long non-coding RNAs found in this study are reported in Supplemental Table 7.

### Identification of CREB-family transcription factors expressed in collecting duct cells

In many cell types, the effects of cyclic AMP on transcription are due to activation of the transcription factor CREB (Gene symbol: *Creb1*). This transcription factor has been proposed to mediate the effects of vasopressin on gene transcription in collecting duct cells^30^,^31^,^32^,^33^. However, recent evidence raises doubts about the role of Creb1 in the vasopressin response in mpkCCD cells^34^. Creb1 is a member of the b-ZIP transcription factor family. Table 3 shows the relative abundances of the b-ZIP transcription factor mRNAs found in vehicle-treated and dDAVP-treated mpkCCD cells in this study. Note that the expression level of *Creb1* is very low (TPM = 0.42) compared to a number of other cAMP-regulated b-ZIP transcription factors, suggesting the possibility that more abundant Creb-like transcription factors, such as Atf4 or Creb3, could be involved in the transcriptional effects of vasopressin. Among the b-ZIP transcription factors reported in Table 3, only Jun exhibited a coordinate change in mRNA abundance and RNA polymerase II biding (Table 2); both measures were decreased. Previous studies have demonstrated the vasopressin decreases c-Jun phosphorylation at Ser73 in mpkCCD cells^35^,^36^ and an AP-1 (Fos/Jun) binding site has been documented in the promotor region of the *Aqp2* gene^33^.

## Discussion

In this paper, we use the methods of systems biology to investigate mechanisms involved in the long-term regulation of the abundance of the water channel aquaporin-2 by the peptide hormone vasopressin. Given that levels of aquaporin-2 protein and mRNA are markedly increased by vasopressin^4^,^14^,^15^, it appears that this vasopressin response is mediated by either an increase in aquaporin-2 mRNA stability or an increase in *Aqp2* gene transcription. The evidence presented in this paper, showing that the vasopressin-induced increase in aquaporin-2 mRNA is accompanied by a marked increase in the binding of RNA polymerase II to the *Aqp2* gene body (Fig. 2A), strongly supports the latter possibility, i.e. transcriptional regulation. Obviously, the two mechanisms are not mutually exclusive. However, the analysis of the 3′-UTR of the aquaporin-2 mRNA in this paper, did not reveal evidence of an AU-rich element required for mRNA stability control, consistent with observations in prior studies in mpkCCD cells^16^,^17^.

One of the chief observations in this study is that, although vasopressin appears to regulate the transcription of several genes in mpkCCD cells, the increases in aquaporin-2 mRNA level and RNA polymerase II binding to the *Aqp2* gene far outstrip changes for all other genes (Fig. 5). This implies that the vasopressin-dependent transcriptional regulation has a high degree of selectivity for *Aqp2*. The results indicate that the selectivity occurs through regulation of transcriptional elongation (Fig. 4). In biology, such selectivity generally implies regulation by multiple factors. For example, it may involve several transcription factors that must be activated together^37^. Although a single transcription factor may bind to promotors or enhancers of multiple genes, coincident binding of three or four transcription factors is more likely to be unique to a single gene. Thus, Creb1 (or another Creb-like cAMP responsive TF) is unlikely to alone be responsible for regulation of *Aqp2* gene expression, contrary to what is commonly assumed in review articles about vasopressin regulation^3^,^30^,^32^. Had we, instead, found a more widespread regulation of genes in response to vasopressin, it would seem more likely that a single transcription factor could have been responsible. Such may be the case with vasopressin-mediated control of transcriptional initiation, which was associated with increased RNA polymerase II binding to the promoter proximal region of most expressed genes (Fig. 2D).

The foregoing argument can be formalized in terms of information theory^38^ and Shannon information content^18^. The amount of information needed to specify one gene (*Aqp2*) to be regulated by vasopressin out of 24000 protein coding genes is log_2_(24000/1) = 14.6 bits. In contrast, if we consider that 35 genes out of 24000 are regulated (Tables 1 and 2), the information content required would be log_2_(24000/35) = 9.4 bits. Or if the regulation were less selective, e.g. if one-third of genes were regulated, the required information content would be only log_2_(24000/8000) = 1.6 bits. If we assume that the information necessary for the regulation is conveyed is via transcription factor binding, more transcription factor binding sites would evidently be needed for selective regulation than for widespread regulation. Specifically, according to O’Neill *et al*.^38^, the information conveyed by binding of a particular transcription factor is log_2_(G/M) where G is the number of protein coding genes and M is the number of high affinity binding sites. For most transcription factors, M is typically at least 1000. (For example, Creb1 binding in hepatocytes targets promoters of more than 4000 genes^39^). Thus, if the typical transcription factor targets 1000 out of 24000 genes, for example, the amount of information conveyed would be log_2_(24000/1000) or 4.5 bits. Thus, the binding of at least four independent transcription factors would be required to selectively regulate the *Aqp2* gene alone (14.6 bits of information), and at least two independent transcription factors would be required to selectively regulate 35 out of 24000 genes (9.4 bits). We conclude then, that the selective transcriptional regulation demonstrated in this paper, implies that transcriptional regulation of *Aqp2* must involve multiple transcription factors that bind at independent sites.

There are multiple putative transcription factor binding sites in the promotor region that could play roles in regulation of *Aqp2* gene transcription^10^. Analysis of the 5′-flanking regions of the *Aqp2* gene from several species identified several conserved binding motifs that play putative roles in transcriptional regulation^10^,^40^,^41^,^42^, including a cAMP response element (CRE), a homeobox (HOX) site, a GATA site, several ETS sites, an Sp1 site, a nuclear factor of activated T cells (NFAT) site, a Forkhead box (FOX) site and a retinoid X receptor (RXR) site. In addition, a putative AP-1 binding site has been reported^33^. Furthermore, a putative site for Kruppel-like factor (Klf) binding has been identified in the first intron of the *Aqp2* gene^42^. Identification of the TFs that bind to these sites will likely require a combination of ChIP-seq studies using TF-specific antibodies and TF gene deletions with genome editing techniques. Proteomics studies revealed three transcription factors that underwent translocation to the nucleus in mpkCCD cells in response to dDAVP, namely, JunD, Elf3, and Gatad2b^43^. These TFs potentially bind to the AP-1 site, the ETS site, and the GATA site of the *Aqp2* gene, respectively, and are therefore candidates for roles in vasopressin-mediated transcriptional regulation.

Previous studies in other cell types have demonstrated that most active genes manifest a predominance of RNA polymerase II binding to the PPR^21^, and therefore our finding of this pattern in mpkCCD cells (Figs 2D and 4B) is far from unique. The pattern has been attributed to the phenomenon of *promotor proximal pausing* (Fig. 4A), a halt in transcriptional elongation within a few hundred bp of the transcriptional initiation site^44^. Promoter proximal pausing of RNA polymerase II was originally described in *Drosophila* heat shock gene (*HSP70*) transcription^45^. Pausing occurs in most genes transcribed by RNA polymerase II. Promotor proximal pausing appears to play an important role in both transcriptional regulation and quality control^46^.

An interesting aspect of this study is the demonstration that vasopressin increases RNA polymerase II binding to the promotor-proximal region of a majority of expressed genes (Figs 2D and 4B), even though few of these show increases in RNA polymerase II binding throughout the gene body. This finding suggests that there is widespread positive regulation of transcriptional *initiation* among expressed genes. Recent investigations have shown that certain transcription factors, most notably Myc, have actions that are not selective for specific genes, but rather act as general amplifiers that accelerate the transcription of virtually all expressed genes^47^. (Myc mRNA abundance was nominally increased in response to vasopressin in this study by 60% [https://helixweb.nih.gov/ESBL/Database/Vasopressin/]). Identification of genomic binding sites for Myc in collecting duct cells has, however, not yet been reported.

## Methods

### Cell Culture

All experiments were performed in mpkCCD11 cells as previously described Briefly, cells were expanded to ~80% confluence on 25-cm2 plastic flasks (Corning), trypsinized (0.05% trypsin, 1.5 mM EDTA) and resuspended in 10 ml DMEM/F12, and then seeded on permeable supports (75-mm2 diameter, 0.4-μm pore size, Corning) at a ratio of 1:10 and grown in 1:1 DMEM/F12 (Invitrogen) containing 2% fetal bovine serum, insulin, dexamethasone, triiodothyronine, epidermal growth factor, selenium, and transferrin as previously described^10^. Cells were transferred to permeable supports (75-mm2 diameter, 0.4-μm pore size, Corning), and grown until confluency as documented by transepithelial resistance measurements (Epithelial Volt ohmmeter; WPI) (RTE) of ≥5 KΩ ∙ cm2). At that point, the serum was removed, and cells were maintained for one more day in serum-free medium. Serum was removed and the V2 receptor-selective vasopressin analog dDAVP (0.1 nM) or its vehicle was added for the final 24 hours before cell harvest.

### Immunoblotting

Samples were diluted in Laemmli buffer (10 mM Tris, pH 6.8, 1.5% SDS, 6% glycerol, 0.05% bromophenol blue, and 40 mM dithiothreitol) and subjected to SDS-PAGE. Immunoblot analysis using nitrocellulose membranes was performed as described previously^48^. Both blocking buffer and infrared dye-coupled secondary antibodies were obtained from LI-COR (Lincoln, NE). Fluorescence signals from discrete bands were read out using the LI-COR Odyssey System. The rabbit polyclonal anti-AQP2 antibody used 1:2000 was described in Hoffert *et al*.^48^. The phosphospecific antibody recognizing phosphorylated Ser5 present in heptad repeats in the COOH-terminal domain of Polr2a was purchased from Abcam (ab5131) and used at 1:1000 following the manufacturer’s protocol. Bands were quantified by densitometry.

Samples were diluted in Laemmli buffer and subjected to SDS-PAGE as previously described^10^. Immunoblot analysis using nitrocellulose membranes was performed as previously described^10^. The anti-AQP2 antibody (1:2000) was from Hoffert *et al*.^48^. A rabbit polyclonal phosphospecific antibody recognizing phosphorylated Ser5 present in heptad repeats in the COOH-terminal domain of Polr2a was purchased from Abcam (ab5131) and used at 1:1000 following the manufacturer’s protocol. Bands were quantified by densitometry.

### Immunofluorescence Microscopy

Immunofluorescence labeling was done as described^11^. The anti-AQP2 antibody (described above) was used at 1:500. Confocal fluorescence micrographs were obtained using a Zeiss LSM 510 microscope (Carl Zeiss; NHLBI, Light Microscopy Core Facility).

### Deep Sequencing

**Figure 6:**
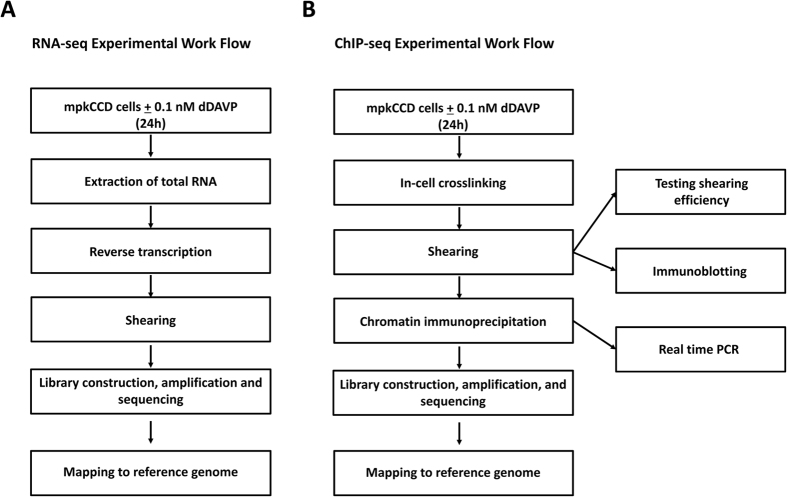
Summary of experimental workflows for RNA-seq (A) and ChIP-seq for the large subunit of RNA polymerase II (B). ChIP (chromatin immunoprecipitation) used an antibody to the large subunit of RNA Polymerase II (Gene Symbol: *Polr2a*). Boxes at right summarize experiments performed for quality control in ChIP-seq. See text for details.

mpkCCD cells were grown in the presence or absence of dDAVP (0.1 nM for 24 hrs) for RNA-seq or ChIP-seq experiments. Procedures for RNA-seq are summarized in Fig. 6 (left). Total RNA was isolated using TRIZOL and QIAGEN RNeasy Mini (QIAGEN). Reverse transcription with an oligo-dT primer and cDNA amplification were done following a small-sample RNA-seq protocol modified from Lee *et al*.^49^, based on single-cell methods^50^. The libraries were made using an Ovation Ultralow Library System (NuGen). cDNAs ranging from 200 to 400 bp were selected on 2% agarose gel and sequenced on a HiSeq2000 platform (Illumina) to generate 50-bp paired-end FASTQ sequences. The raw FASTQ sequences were mapped to the mouse reference genome (mm10) using STAR 2.3.0.39. Mapped reads were visualized on the UCSC Genome Browser and the Integrative Genomics Viewer (Broad Institute). The data were normalized by the TPM (Transcripts per Million) method^51^. This normalization is similar to the more commonly used RPKM, normalizing for the length of each mRNA species and the sequencing depth of a sample, but calculated in the opposite order.

Procedures for ChIP-seq are summarized in Fig. 6 (right). After cross-linking (formaldehyde 1.11%), nuclei were isolated and then sheared into approximately 300-bp fragments (Covaris S2 SonoLAB). Samples underwent chromatin immunoprecipitation (SimpleChIP protocol (#9003, Cell Signaling Technology) using an anti-Polr2a antibody (Catalog #: MMS-126R, Covance). After the ChIP step, crosslinking was reversed and samples were processed for library construction. Library construction procedures were identical to those used for RNA-seq. cDNAs ranging from 200 to 400 bp were selected on 2% agarose gel and sequenced on a HiSeq2000 platform (Illumina) to generate 50-bp FASTQ sequences. The raw FASTQ sequences were mapped to the mouse Reference Genome (mm10) using the Burrows-Wheeler Aligner (*BWA)*. Mapped reads were visualized on the UCSC Genome Browser and the Integrative Genomics Viewer (Broad Institute).

## Additional Information

**How to cite this article**: Sandoval, P. C. *et al*. Systems-level analysis reveals selective regulation of *Aqp2* gene expression by vasopressin. *Sci. Rep*. **6**, 34863; doi: 10.1038/srep34863 (2016).

## Supplementary Material

Supplementary Information

Supplementary Table S1

Supplementary Table S2

Supplementary Table S3

Supplementary Table S4

Supplementary Table S5

Supplementary Table S6

Supplementary Table S7

Supplementary Table S8

## Figures and Tables

**Table 1:** Genes with significant increases in mRNA (RNA-seq) and RNA polymerase II binding (ChIP-seq) based on Fig. 5.

Gene Symbol	Annotation	RNA Polymerase II Binding, log_2_(dDAVP/Vehicle), n = 3	TPM for mRNA from RNA-seq log_2_(dDAVP/vehicle), n = 9
*Aqp2*	aquaporin 2	2.78 ± 0.46	4.52 ± 0.85
*B3gnt7*	UDP-GlcNAc:betaGal beta-1,3-N-acetylglucosaminyltransferase 7	2.03 ± 0.16	3.55 ± 0.65
*Arl4d*	ADP-ribosylation factor-like 4D	1.49 ± 0.19	2.32 ± 0.82
*Lypd3*	Ly6/Plaur domain containing 3	0.73 ± 0.24	1.76 ± 0.76
*H6pd*	hexose-6-phosphate dehydrogenase glucose 1-dehydrogenase	0.66 ± 0.07	1.49 ± 0.34
*Hilpda*	hypoxia inducible lipid droplet associated	0.91 ± 0.24	1.29 ± 0.42
*Cdk3-ps*	cyclin-dependent kinase 3, pseudogene Cdk3-ps, non-coding RNA	0.76 ± 0.11	1.2 ± 0.49
*Snhg7*	small nucleolar RNA host gene 7 Snhg7, long non-coding RNA	0.56 ± 0.18	1.17 ± 0.71
*1810019D21Rik*	RIKEN cDNA 1810019D21 gene 1810019D21Rik, long non-coding RNA	0.77 ± 0.68	1.09 ± 0.27
*Nr4a1*	nuclear receptor subfamily 4, group A, member 1	1.08 ± 0.18	1.08 ± 0.59
*Nfkbiz*	nuclear factor of kappa light polypeptide gene enhancer in B cells inhibitor, zeta	0.58 ± 0.18	1.08 ± 0.34
*Bhlhe40*	basic helix-loop-helix family, member e40	0.76 ± 0.44	1.07 ± 0.64
*Cdk18*	cyclin-dependent kinase 18	0.68 ± 0.22	1.02 ± 0.41
*Tob1*	transducer of ErbB-2.1	0.56 ± 0.37	1.01 ± 0.43
*Polr3d*	polymerase (RNA III DNA directed)	0.55 ± 0.17	0.97 ± 0.46
*Slc2a4rg-ps*	Slc2a4 regulator, pseudogene Slc2a4rg-ps, non-coding RNA	0.73 ± 0.31	0.96 ± 0.59
*Tspan1*	tetraspanin 1	0.79 ± 0.36	0.95 ± 0.51
*Srxn1*	sulfiredoxin 1 homolog S. cerevisiae	0.60 ± 0.37	0.92 ± 0.40
*Sat1*	spermidine/spermine N1-acetyl transferase 1	1.05 ± 0.30	0.91 ± 0.89
*Zfp750*	zinc finger protein 750	0.54 ± 0.19	0.91 ± 0.58
*Sptbn2*	spectrin beta, non-erythrocytic 2	0.66 ± 0.16	0.85 ± 0.55
*9330133O14Rik*	RIKEN cDNA 9330133O14 gene 9330133O14Rik,	1.30 ± 0.65	0.83 ± 0.48
*Atp1b1*	ATPase, Na+/K+ transporting, beta 1 polypeptide long non-coding RNA	0.59 ± 0.22	0.82 ± 0.47
*Id3*	inhibitor of DNA binding 3	1.64 ± 0.53	0.81 ± 0.46
*Sik1*	salt inducible kinase 1	0.85 ± 0.23	0.81 ± 0.58
*Marveld1*	MARVEL membrane-associating domain containing 1	0.66 ± 0.35	0.77 ± 0.34
*Trmt5*	TRM5 tRNA methyltransferase 5	0.56 ± 0.20	0.72 ± 0.75
*Osgin1*	oxidative stress induced growth inhibitor 1	0.79 ± 0.15	0.71 ± 0.59
*Krt19*	keratin 19	0.97 ± 0.29	0.62 ± 0.36

Values are mean ± standard error for the indicated number of replicates.

**Table 2:** Genes with significant decreases in mRNA (RNA-seq) and RNA polymerase II binding (ChIP-seq) from Fig. 5.

Gene Symbol	Protein Name	RNA Polymerase II Binding, log_2_(dDAVP/Vehicle), n = 3	TPM for mRNA from RNA-seq log_2_(dDAVP/vehicle), n = 9
*Gadd45b*	growth arrest and DNA-damage-inducible 45 beta	−0.62 ± 0.25	−0.63 ± 0.34
*Coa6*	cytochrome c oxidase assembly factor 6	−0.58 ± 0.3	−0.89 ± 0.8
*Selm*	selenoprotein M	−0.58 ± 0.37	−0.92 ± 0.69
*Rasl11b*	RAS-like, family 11, member B	−0.73 ± 0.14	−1.02 ± 0.55
*Cebpzos*	CCAAT/enhancer binding protein C/EBP, zeta, opposite strand	−0.55 ± 0.15	−1.23 ± 0.79
*Jun*	jun proto-oncogene	−0.61 ± 0.6	−1.53 ± 0.41

Values are mean ± standard error for the indicated number of replicates.

**Table 3:** b-ZIP transcription factors expressed in mpkCCD (clone 11) cells.

Gene Symbol	Annotation	mean TPM (vehicle)	mean TPM (dDAVP)	log2 (dDAVP/Vehicle) [mean ± SE]
Atf4	cyclic AMP-dependent transcription factor ATF-4	301.19	386.41	0.10 ± 0.31
Junb	transcription factor jun-B	158.29	50.80	−1.04 ± 0.68
Nfe2l2	nuclear factor erythroid 2-related factor 2	34.20	48.72	0.57 ± 0.73
Jund	transcription factor jun-D	210.61	42.77	−1.24 ± 0.66
Atf6b	cyclic AMP-dependent transcription factor ATF-6 beta	14.14	16.35	−0.53 ± 0.65
Nfe2l1	nuclear factor erythroid 2-related factor 1	8.36	14.25	0.34 ± 0.4
Creb3	cyclic AMP-responsive element-binding protein 3	7.06	11.61	0.15 ± 0.59
Tef	thyrotroph embryonic factor	2.83	7.43	1.00 ± 0.41
Atf5	cyclic AMP-dependent transcription factor ATF-5	5.38	7.31	0.20 ± 0.86
Mafg	transcription factor MafG	3.33	5.76	−0.15 ± 0.6
Atf1	cyclic AMP-dependent transcription factor ATF-1	5.74	5.28	−0.59 ± 0.42
Maff	transcription factor MafF	3.54	5.17	0.12 ± 0.37
Atf3	cyclic AMP-dependent transcription factor ATF-3	7.25	4.81	−1.65 ± 0.62
Mafk	transcription factor MafK	2.83	2.97	−0.64 ± 0.63
Fos	proto-oncogene c-Fos	2.53	2.76	−0.10 ± 0.66
Dbp	D site-binding protein	3.91	2.47	−0.17 ± 0.62
Jun	transcription factor AP-1	5.57	2.28	−1.53 ± 0.41
Crebzf	CREB/ATF bZIP transcription factor	2.68	1.46	−0.47 ± 0.53
Crem	cAMP-responsive element modulator	0.81	1.39	1.42 ± 0.72
Nfe2l3	nuclear factor erythroid 2-related factor 3	2.47	0.92	−0.72 ± 0.91
Atf6	cyclic AMP-dependent transcription factor ATF-6 alpha	0.29	0.76	0.85 ± 0.79
Fosl2	fos-related antigen 2	0.59	0.70	0.59 ± 0.55
Atf2	cyclic AMP-dependent transcription factor ATF-2	0.46	0.58	−0.34 ± 0.72
Creb1	cyclic AMP-responsive element-binding protein 1	0.42	0.36	−0.53 ± 1.02

## References

[b1] Nielsen S. . Vasopressin increases water permeability of kidney collecting duct by inducing translocation of aquaporin-CD water channels to plasma membrane. Proceedings of the National Academy of Sciences, USA 92, 1013–1017 (1995). 10.1073/pnas.92.4.1013 PMC42627 7532304

[b2] Sabolic I., Katsura T., Verbabatz J. M. & Brown D. The AQP2 water channel: effect of vasopressin treatment, microtubule disruption, and distribution in neonatal rats. Journal of Membrane Biology 143, 165–177 (1995). 10.1007/BF00233445 7539496

[b3] Nielsen S. . Aquaporins in the kidney: from molecules to medicine. Physiol Rev. 82, 205–244 (2002). 11773613 10.1152/physrev.00024.2001

[b4] Hasler U. . Long term regulation of aquaporin-2 expression in vasopressin-responsive renal collecting duct principal cells. J Biol. Chem. 277, 10379–10386 (2002). 11782489 10.1074/jbc.M111880200

[b5] DiGiovanni S. R., Nielsen S., Christensen E. I. & Knepper M. A. Regulation of collecting duct water channel expression by vasopressin in Brattleboro rat. Proceedings of the National Academy of Sciences, USA 91, 8984–8988 (1994). 10.1073/pnas.91.19.8984 PMC44731 7522327

[b6] Knepper M. A. Systems biology in physiology: the vasopressin signaling network in kidney. Am. J. Physiol Cell Physiol 303, C1115–C1124 (2012). 22932685 10.1152/ajpcell.00270.2012 PMC3530773

[b7] Mimura I., Kanki Y., Kodama T. & Nangaku M. Revolution of nephrology research by deep sequencing: ChIP-seq and RNA-seq. Kidney Int. 85, 31–38 (2014). 23986147 10.1038/ki.2013.321

[b8] Hawkins R. D., Hon G. C. & Ren B. Next-generation genomics: an integrative approach. Nat. Rev. Genet. 11, 476–486 (2010). 20531367 10.1038/nrg2795 PMC3321268

[b9] Chassin C., Bens M. & Vandewalle A. Transimmortalized proximal tubule and collecting duct cell lines derived from the kidneys of transgenic mice. Cell Biol. Toxicol. 23, 257–266 (2007). 17219250 10.1007/s10565-006-0169-y

[b10] Yu M. J. . Systems-level analysis of cell-specific AQP2 gene expression in renal collecting duct. Proc. Natl. Acad. Sci. USA 106, 2441–2446 (2009). 19190182 10.1073/pnas.0813002106 PMC2650175

[b11] Sandoval P. C. . Proteome-wide measurement of protein half-lives and translation rates in vasopressin-sensitive collecting duct cells. J. Am. Soc. Nephrol. 24, 1793–1805 (2013). 24029424 10.1681/ASN.2013030279 PMC3810089

[b12] Nedvetsky P. I. . Reciprocal regulation of aquaporin-2 abundance and degradation by protein kinase A and p38-MAP kinase. J Am Soc. Nephrol. 21, 1645–1656 (2010). 20724536 10.1681/ASN.2009111190 PMC3013543

[b13] Fujita N. . Role of water channel AQP-CD in water retention in SIADH and cirrhotic rats. American Journal of Physiology 269, F926–F931 (1995). 8594889 10.1152/ajprenal.1995.269.6.F926

[b14] Ecelbarger C. A. . Role of renal aquaporins in escape from vasopressin-induced antidiuresis in rat. Journal of Clinical Investigation 99, 1852–1863 (1997). 9109429 10.1172/JCI119352 PMC508009

[b15] Hayashi M. . Role of vasopressin V2 receptor in acute regulation of aquaporin-2. Kidney Blood Press Res. 19, 32–37 (1996). 8818115 10.1159/000174043

[b16] Hasler U., Leroy V., Martin P. Y. & Feraille E. Aquaporin-2 Abundance in the Renal Collecting Duct: New Insights from Cultured Cell Models. Am. J. Physiol Renal Physiol (2009). 10.1152/ajprenal.00053.2009 19244407

[b17] Hasler U., Nielsen S., Feraille E. & Martin P. Y. Posttranscriptional control of aquaporin-2 abundance by vasopressin in renal collecting duct principal cells. Am J Physiol Renal Physiol 290, F177–F187 (2006). 15985652 10.1152/ajprenal.00056.2005

[b18] Shannon C. E. A Mathematical Theory of Communication. Bell System Technical Journal 27, 379–423 (1948).

[b19] Khositseth S. . Quantitative protein and mRNA profiling shows selective post-transcriptional control of protein expression by vasopressin in kidney cells. Mol. Cell Proteomics. 10, M110 (2011). 10.1074/mcp.M110.004036 PMC3013460 20940332

[b20] Lee T. I. & Young R. A. Transcriptional regulation and its misregulation in disease. Cell 152, 1237–1251 (2013). 23498934 10.1016/j.cell.2013.02.014 PMC3640494

[b21] Liu X., Kraus W. L. & Bai X. Ready, pause, go: regulation of RNA polymerase II pausing and release by cellular signaling pathways. Trends Biochem. Sci. 40, 516–525 (2015). 26254229 10.1016/j.tibs.2015.07.003 PMC4553066

[b22] Hsin J. P. & Manley J. L. The RNA polymerase II CTD coordinates transcription and RNA processing. Genes Dev. 26, 2119–2137 (2012). 23028141 10.1101/gad.200303.112 PMC3465734

[b23] Ibrahim H., Lee Y. J. & Curthoys N. P. Renal response to metabolic acidosis: role of mRNA stabilization. Kidney Int. 73, 11–18 (2008). 17914349 10.1038/sj.ki.5002581 PMC2814166

[b24] Robert-Nicoud M. . Transcriptome of a mouse kidney cortical collecting duct cell line: effects of aldosterone and vasopressin. Proc. Natl. Acad. Sci. USA 98, 2712–2716 (2001). 11226305 10.1073/pnas.051603198 PMC30204

[b25] Cai Q. . Vasopressin receptor subtype 2 activation increases cell proliferation in the renal medulla of AQP1 null mice. Am. J. Physiol Renal Physiol 293, F1858–F1864 (2007). 17913837 10.1152/ajprenal.00068.2007

[b26] Sanduja S., Blanco F. F. & Dixon D. A. The roles of TTP and BRF proteins in regulated mRNA decay. Wiley. Interdiscip. Rev. RNA 2, 42–57 (2011). 21278925 10.1002/wrna.28 PMC3030256

[b27] Miller R. L., Sandoval P. C., Pisitkun T., Knepper M. A. & Hoffert J. D. Vasopressin inhibits apoptosis in renal collecting duct cells. Am J Physiol Renal Physiol (2012). 10.1152/ajprenal.00431.2012 PMC3543627 23136001

[b28] Dey B. K., Mueller A. C. & Dutta A. Long non-coding RNAs as emerging regulators of differentiation, development, and disease. Transcription 5, e944014 (2014). 25483404 10.4161/21541272.2014.944014 PMC4581346

[b29] Nelson B. R. . A peptide encoded by a transcript annotated as long noncoding RNA enhances SERCA activity in muscle. Science 351, 271–275 (2016). 26816378 10.1126/science.aad4076 PMC4892890

[b30] Pearce D. . Collecting duct principal cell transport processes and their regulation. Clin. J. Am. Soc. Nephrol. 10, 135–146 (2015). 24875192 10.2215/CJN.05760513 PMC4284417

[b31] Kortenoeven M. L. & Fenton R. A. Renal aquaporins and water balance disorders. Biochim. Biophys. Acta 1840, 1533–1549 (2014). 24342488 10.1016/j.bbagen.2013.12.002

[b32] Marples D., Frokiaer J. & Nielsen S. Long-term regulation of aquaporins in the kidney. Am. J. Physiol 276, F331–F339 (1999). 10070156 10.1152/ajprenal.1999.276.3.F331

[b33] Yasui M., Zelenin S. M., Celsi G. & Aperia A. Adenylate cyclase-coupled vasopressin receptor activates AQP2 promoter via a dual effect on CRE and AP1 elements. Am J Physiol Renal Physiol 272, F443–F450 (1997). 10.1152/ajprenal.1997.272.4.F443 9140044

[b34] Kortenoeven M. L. . In mpkCCD cells, long-term regulation of aquaporin-2 by vasopressin occurs independent of protein kinase A and CREB but may involve Epac. Am J Physiol Renal Physiol 302, F1395–F1401 (2012). 22419689 10.1152/ajprenal.00376.2011

[b35] Bolger S. J. . Quantitative Phosphoproteomics in Nuclei of Vasopressin-Sensitive Renal Collecting Duct Cells. Am J Physiol Cell Physiol 303, C1006–C1020 (2012). 22992673 10.1152/ajpcell.00260.2012 PMC3492837

[b36] Rinschen M. M. . Quantitative phosphoproteomic analysis reveals vasopressin V2-receptor-dependent signaling pathways in renal collecting duct cells. Proc. Natl. Acad. Sci. USA 107, 3887 (2010). 10.1073/pnas.0910646107 PMC2840509 20139300

[b37] Busser B. W., Bulyk M. L. & Michelson A. M. Toward a systems-level understanding of developmental regulatory networks. Curr. Opin. Genet. Dev. (2008). 10.1016/j.gde.2008.09.003 PMC2704888 18848887

[b38] O’Neill P. K., Forder R. & Erill I. Informational requirements for transcriptional regulation. J. Comput. Biol. 21, 373–384 (2014). 24689750 10.1089/cmb.2014.0032 PMC4010175

[b39] Everett L. J. . Integrative genomic analysis of CREB defines a critical role for transcription factor networks in mediating the fed/fasted switch in liver. BMC. Genomics 14, 337 (2013). 23682854 10.1186/1471-2164-14-337 PMC3671974

[b40] Uchida S., Matsumura Y., Rai T., Sasaki S. & Marumo F. Regulation of aquaporin-2 gene transcription by GATA-3. Biochem. Biophys. Res. Commun. 232, 65–68 (1997). 9125153 10.1006/bbrc.1997.6236

[b41] Rai T., Uchida S., Marumo F. & Sasaki S. Cloning of rat and mouse aquaporin-2 gene promoters and identification of a negative cis-regulatory element. Am J Physiol 273, F264–F273 (1997). 9277587 10.1152/ajprenal.1997.273.2.F264

[b42] Tchapyjnikov D. . Proteomic profiling of nuclei from native renal inner medullary collecting duct cells using LC-MS/MS. Physiol Genomics 40, 167–183 (2010). 19996160 10.1152/physiolgenomics.00148.2009 PMC2825761

[b43] Schenk L. K. . Quantitative proteomics identifies vasopressin-responsive nuclear proteins in collecting duct cells. J Am Soc. Nephrol. 23, 1008–1018 (2012). 22440904 10.1681/ASN.2011070738 PMC3358758

[b44] Rahl P. B. . c-Myc regulates transcriptional pause release. Cell 141, 432–445 (2010). 20434984 10.1016/j.cell.2010.03.030 PMC2864022

[b45] Gilmour D. S. & Lis J. T. RNA polymerase II interacts with the promoter region of the noninduced hsp70 gene in Drosophila melanogaster cells. Mol. Cell Biol. 6, 3984–3989 (1986). 3099167 10.1128/mcb.6.11.3984-3989.1986 PMC367162

[b46] Jonkers I. & Lis J. T. Getting up to speed with transcription elongation by RNA polymerase II. Nat. Rev. Mol. Cell Biol. 16, 167–177 (2015). 25693130 10.1038/nrm3953 PMC4782187

[b47] Wolf E., Lin C. Y., Eilers M. & Levens D. L. Taming of the beast: shaping Myc-dependent amplification. Trends Cell Biol. 25, 241–248 (2015). 25475704 10.1016/j.tcb.2014.10.006 PMC4380620

[b48] Hoffert J. D. . Vasopressin-stimulated increase in phosphorylation at Ser269 potentiates plasma membrane retention of aquaporin-2. J Biol. Chem. 283, 24617–24627 (2008). 18606813 10.1074/jbc.M803074200 PMC2528999

[b49] Lee J. W., Chou C. L. & Knepper M. A. Deep sequencing in microdissected renal tubules Identifies nephron segment-specific transcriptomes. J. Am. Soc. Nephrol. 26, 2669–2677 (2015). 25817355 10.1681/ASN.2014111067 PMC4625681

[b50] Tang F. . RNA-Seq analysis to capture the transcriptome landscape of a single cell. Nat. Protoc. 5, 516–535 (2010). 20203668 10.1038/nprot.2009.236 PMC3847604

[b51] Wagner G. P., Kin K. & Lynch V. J. Measurement of mRNA abundance using RNA-seq data: RPKM measure is inconsistent among samples. Theory. Biosci. 131, 281–285 (2012). 22872506 10.1007/s12064-012-0162-3

