# Supplementary material for: Systems-level analysis reveals selective regulation of Aqp2 gene expression by vasopressin
Source: Sci Rep. 2016 Oct 11;6:34863. doi: 10.1038/srep34863 (PMC5057153; doi:10.1038/srep34863)
Supplement: Supplementary Information [file srep34863-s1.pdf]

Supplementary information for

Systems-level analysis reveals selective regulation of *Aqp2* gene expression by vasopressin

Pablo C. Sandoval, J'Neka S. Claxton, Jae Wook Lee*,
Fahad Saeed, Jason D. Hoffert, and Mark A. Knepper‡

Epithelial Systems Biology Laboratory

NHLBI, National Institutes of Health,

Bethesda, MD 20892-1603, USA

and

***National Cancer Center of Korea**

Goyang-si Gyeonggi-do, Korea

Running title: **Transcriptional Regulation of *Aqp2* Gene**

‡Correspondence to Mark A. Knepper, National Institutes of Health, Bldg. 10, Room 6N307, 10 CENTER DR, MSC-1603, Bethesda, MD 20892-1603, Phone: (301) 496-3064, Fax: (301) 402-1443, E-mail:knepperm@nhlbi.nih.gov

Index to Supplementary Materials for Sandoval et al. "Systems-level analysis reveals selective regulation of Aqp2 gene expression by vasopressin"

Supplementary Figure 1. See below or click here.

Materials_and_Methods.docx See below or click here.

Supplementary_Table_1.xlsx See separate file or click here.

Supplementary_Table_2.xlsx See separate file or click here.

Supplementary_Table_3.xlsx See separate file or click here.

Supplementary_Table_4.xlsx See separate file or click here.

Supplementary_Table_5.xlsx See separate file or click here.

Supplementary_Table_6.xlsx See separate file or click here.

Supplementary_Table_7.xlsx See separate file or click here.

Supplementary_Table_8.xlsx See separate file or click here.

Supplementary Figure 1. Distribution of reads mapping to Aqp2 gene shown with linear scaling. See main text for log scaling.

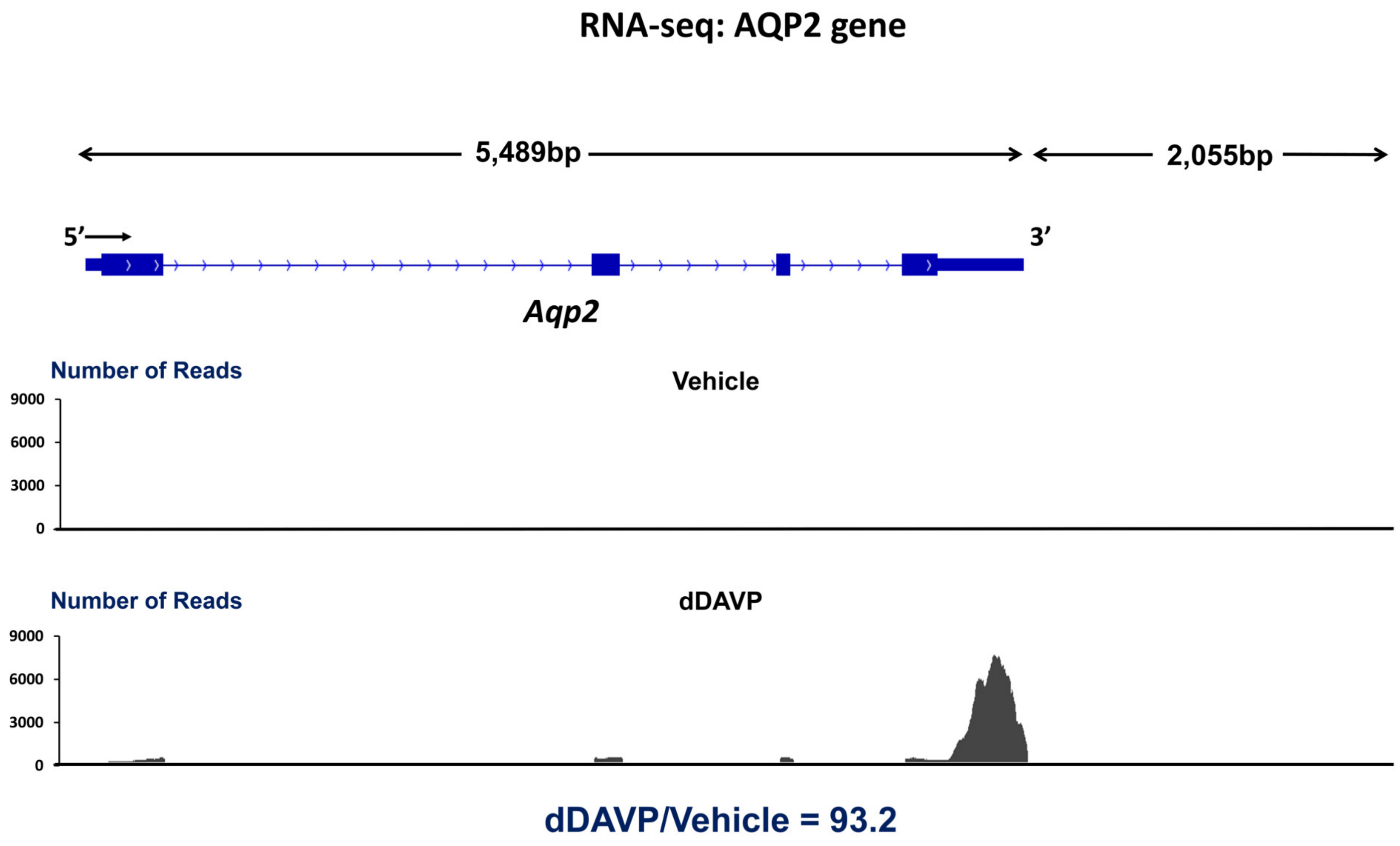

Materials and Methods

Cell culture

All experiments were performed in mpkCCD11 cells as previously described [1]. Briefly, cells were expanded to ~80% confluence on 25-cm2 plastic flasks (Corning), trypsinized (0.05% trypsin, 1.5 mM EDTA) and resuspended in 10 ml DMEM/F12, and then seeded on permeable supports (75-mm2 diameter, 0.4-µm pore size, Corning) at a ratio of 1:10 and grown in 1:1 DMEM/F12 (Invitrogen) containing 2% fetal bovine serum, insulin, dexamethasone, triiodothyronine, epidermal growth factor, selenium, and transferrin as previously described [2]. Once cells reached confluence (~x 106 cells) and demonstrated a transepithelial resistance (RTE) of ≥5 KΩ•cm2, they were maintained in serum-free medium for 24 h prior to treatment with or without 0.1 nM dDAVP in serum-free medium for 24 h.

Immunoblotting

Samples were diluted in Laemmli buffer (10 mM Tris, pH 6.8, 1.5% SDS, 6% glycerol, 0.05% bromophenol blue, and 40 mM dithiothreitol) and subjected to SDS-PAGE. Immunoblot analysis using nitrocellulose membranes was performed as described previously. [3] Both blocking buffer and infrared dye-coupled secondary antibodies were obtained from LI-COR (Lincoln, NE). Fluorescence signals from discrete bands were read out using the LI-COR Odyssey System. The rabbit polyclonal anti-AQP2 antibody used 1:2000 was described in Hoffert et al. [4].The phosphospecific antibody recognizing phosphorylated Ser5 present in 51 heptad repeats in the COOH-terminal domain of Polr2a was purchased from Abcam (ab5131) and used at 1:1000 following the manufacturer's protocol. Bands were quantified by densitometry.

Immunofluorescence Microscopy

Immunofluorescence labeling was done as described [5]. The anti-AQP2 antibody (described above) was used at 1:500. Confocal fluorescence micrographs were obtained using a Zeiss LSM 510 microscope (Carl Zeiss; NHLBI, Light Microscopy Core Facility).

RNA-seq

Total DNA-free RNAs were isolated using TRIZOL and QIAGEN RNeasy Mini (QIAGEN). cDNA libraries were created using an RNA-seq protocol originally developed for single-cell transcriptome analysis [6]. 200 ng of total RNAs were used for reverse transcription. After two rounds of PCR, cDNAs ranging 100 ~ 3,000 bp were selected on 2% E-gel (Life Technologies) and purified for shearing. 500 ng of cDNAs were sheared by sonication using Covaris S2 (Covaris) to generate ~200 base-pair fragments. Sheared cDNAs were made into adapter-ligated cDNA libraries using a Mondrian SP+ microfluidic machine and Ovation SP+ UltraLow library systems (NuGen). The cDNA libraries were sequenced in paired-end protocol using HiSeq 2000 sequencer (Illumina). The raw FASTQ sequences were inspected and nucleotides with mapping quality score less than 30 were trimmed using Trimmomatic 0.3.2 [7]. FASTQ sequences that passed this test were mapped to mouse reference genome (mm10) using Spliced Transcripts Alignment to a Reference (STAR) version 2.3.0 [8]. Genomic index for mouse was built from FASTA sequences of mouse chromosomes downloaded from the UCSC table browser website, and the FASTQ sequences were mapped to the mouse reference genome using the following command

STAR --genomeDir <genomedirectory> --readFilesIn <file1.fastq> <file2.fastq> --runThreadN 8 --outFilterMismatchNmax 3 --genomeLoad LoadAndKeep --outSAMstrandField intronMotif --alignIntronMax 10000 --alignMatesGapMax 10000 --outFilterIntronMotifs

Only uniquely mapped reads were included in downstream analysis. The numbers of mapped reads and reads per kilobase exons per million mapped reads (RPKMs) or transcript per million (TPM) for RefSeq transcripts were calculated using HOMER software package [9]. Mapped reads were visualized on the UCSC Genome Browser and Integrative Genomics Viewer (IGV).

ChIP-seq

Confluent monolayers mpkCCD cells were washed in ice-cold 1X PBS (Ca+2/Mg+2-free) followed by cross-linking in 1.11% formaldehyde in Covaris Fixing Buffer (5.5 ml) for 5min at room temperature with gentle rocking (PN 520075, Covaris, Woburn, Massachusetts). Cross-linking was terminated by the addition of 0.3 ml of 1X Covaris Quenching Buffer with rocking. The buffer solution was aspirated and 1X ice-cold PBS was added to each plate. Cells were scraped into 15ml conical tubes, spun down (200 x g, 5 min), and washed twice with PBS. After the second wash, cell pellets were resuspended in 10 ml of 1X Covaris Lysis Buffer for 10 min at 4° C with rocking. Nuclei were pelleted by spinning at 1,700 x g for 5 min at 4° C. Nuclear pellets was resuspended in the 1X Covaris Wash Buffer and incubated for 10 mins at 4° C with rocking. Samples were spun at 1,700 x g for 5 min at 4° C. Nuclear pellets were washed twice with the Covaris Non-ionic Shearing Buffer and spun at 1,700 x g for 5 min at 4° C. Nuclei were resuspended in Covaris Non-ionic Shearing Buffer (Maximum of 1-3x 10^7cells per ml of buffer). <u>Shearing.</u> Two 20 ul aliquots of the resuspended pellets were removed prior to shearing to be used as subsequent unsheared controls for immunoblotting and agarose gel electrophoresis. The remaining samples were transferred to AFA tubes (TC12 x 12 mm) and volumes adjusted to 1 ml (V)). Samples were sheared using a Covaris S2 SonoLAB Single system for two minutes (Duty cycle: 5%; Intensity: 4; cycle: 200). Total nuclear protein concentration of each sample in mcg/ml (R) was determined with the BCA Protein Assay Kit (Thermo Scientific, Rockford, IL). To test shearing efficiency, a 20 ul aliquot from each tube was removed after shearing to test the efficacy of shearing. The aliquots, including the unsheared sample, were incubated with 100 ul 1X TE buffer, 10 ul 10% SDS, and RNAse A (10 ug/ul, Cell Signaling Technology, Danvers, Massachusetts) for 30 mins at 37° C. 1 ul proteinase K (20 ug/ul) was added and the samples were incubated overnight at 65° C. The DNA was purified using DNA Clean & Concentrator columns (Catalog #: D4013, Zymo Research, Irvine, CA) and ran on an E-gel EX 2% agarose (Invitrogen, Carlsbad, CA). Target fragment sizes for chromatin immunoprecipitation were in the range of 150-700 bp in length with average fragment size of 300 bp.

<u>Testing epitope integrity (immunoblotting).</u> Total protein content was determined with the BCA Protein Assay Kit (Thermo Scientific, Rockford, IL). Samples were then diluted with sample buffer (5X Laemmli buffer) at 20% (v/v) of the entire sample volume. Proteins were resolved by SDS-PAGE (4-20% polyacrylamide gels, Criterion, Bio-Rad, Hercules, CA) and transferred electrophoretically onto nitrocellulose membranes as previously described (Hwang, 2010). Membranes were probed with anti-RNA polymerase II (Catalog #: MMS-126R, Covance). The antibody was used at a 1:500 dilution (anti-RNA polymerase II) (in Odyssey blocking buffer containing 0.1% Tween 20) overnight at 4°C. After 1-h incubation with secondary antibody (Li-Cor Biosciences 680 anti-rabbit immunoglobulin G or 800 anti-mouse immunoglobulin G; Lincoln, NE) at 1:5000 dilution, sites of antibody-antigen reaction were detected using an Odyssey infrared imager (Li-Cor).

<u>Chromatin immunoprecipitation.</u> 10 ul of sample equivalent to 2% input was removed prior to IP. Chromatin Immunoprecipitation was performed using 30-90 ug of sheared chromatin in 500 uL of 1X Covaris Non-ionic Shearing Buffer with 10 µl anti-RNA polymerase II antibodies. Samples were incubated overnight at 4°C with rotation, followed by addition of ChIP Grade Protein G Magnet Beads (Catalog #:

9006, Cell Signaling Technology) and incubation for 2 h at 4° C with rotation. The beads were washed a total of four times, including three low salt washes followed by one high salt wash for 5 mins/wash at 4° C (SimpleChIP protocol #9003, Cell Signaling Technology). Immunoprecipitated chromatin was eluted from the beads with 150 ul of 1X ChIP Elution Buffer for 30 mins at 65° C in a thermomixer. To reverse crosslinks, 6 ul of 5M NaCl and 2 ul of 20 ug/ul Proteinase K were added to the chromatin followed by incubation at 65° C overnight. DNA was purified according to SimpleChIP protocol #9003, Cell Signaling Technology.

q-PCR. To check the success of the chromatin immunoprecipitation, input and ChIP'ed DNA samples were amplified with SYBR Green PCR Master Mix (Applied Biosystems) on a 7900HT Fast Real-Time PCR System. Primer sets corresponding to Hox9a and actin genes were used as positive controls for both histone and RNA polymerase II IP's.

Library construction, amplification and sequencing. Loaded fixed volume of total eluate. Library construction was performed on a NuGEN instrument using the protocol and reagents outlined in the Ovation SP Ultralow Library Systems kit. Libraries were then enriched via PCR. After amplification, DNA was measured. A fixed amount of the total measured DNA was used for sequencing in an Illumina Hiseq2000 instrument to obtain single read data.

High-throughput sequencing and read mapping. Libraries for Illumina sequencing of the samples were prepared using the the protocol and reagents outlined in the Ovation SP Ultralow Library Systems kit , NuGen Mondrian SP+ (NuGEN, San Carlos, CA) . Samples were sequenced using an Illumina HiSeq2000, obtaining 1- by 50-bp reads. The data have been deposited in NCBI's Gene Expression Omnibus (GEO) (http://www.ncbi.nlm.nih.gov/geo/query/acc.cgi?acc=GSE79584). The sequencing quality was assessed using FastQC. The first 50 nucleotides (nt) of reads was mapped to the mouse genome (mm10 assembly)

Mapping and quantification. The data comes of the sequencer as FASTQ files. To process these we use the following programs and commands. To align the .fastq file to a mm10 mouse genome and create a .sam file, we used BWA (https://sourceforge.net/projects/bio-bwa/files/). A command line example is: *bwa samse mm10.fa sample7.sai sample7.fastq > sample7.sam bwa aln -t 8 mm10.fa sample7.fastq > sample7.sai*

Then, to convert the format file from .sam to .bam and create a index to the .bam files we use Samtools (https://sourceforge.net/projects/samtools/files/). A command line example is: *Samtools view –bhS sample7.sam > sample7.bam, samtools sort V1_mm10.bam V1_mm10.sorted; samtools index V1_mm10.sorted.bam*

Next, to calculate the coverage reads we use Bedtools (http://bedtools.readthedocs.org/en/latest/). A command line example is: *coverageBed -a V1_mm10.bed -b all_genes_refseq_filter.bed > V1_mm10.bed_coverage.txt*

Then, we plot the coverage along the gene body using NGS-Plot in R. The command line is: *ngs.plot.r -G mm10 -R genebody -C C1_mm10.sorted.bam -O C1_mm10.hist.txt -L 2000 -D refseq*

To create bedgraph files to visualize the data in the UCSD Genome Browser, we use Bedtools. A command line example is: *genomeCoverageBed -bg -trackline -scale 1e-6 -i JP001_8_TTCGCT_L007_R1_concatenated.bed -g mm10.chrom.sizes > CT_histoneK27_repl1.bedgraph*

Reference List

1. Yu MJ, Miller RL, Uawithya P, Rinschen MM, Khositseth S, Braucht DW, Chou CL, Pisitkun T, Nelson RD, Knepper MA: Systems-level analysis of cell-specific AQP2 gene expression in renal collecting duct. *Proc Natl Acad Sci U S A* 106:2441-2446, 2009

2. Khositseth S, Pisitkun T, Slentz DH, Wang G, Hoffert JD, Knepper MA, Yu MJ: Quantitative protein and mRNA profiling shows selective post-transcriptional control of protein expression by vasopressin in kidney cells. *Mol Cell Proteomics* 10:M110, 2011

3. Hoffert JD, Fenton RA, Moeller HB, Simons B, Tchapyjnikov D, McDill BW, Yu MJ, Pisitkun T, Chen F, Knepper MA: Vasopressin-stimulated Increase in Phosphorylation at Ser269 Potentiates Plasma Membrane Retention of Aquaporin-2. *Journal of Biological Chemistry* 283:24617-24627, 2008

4. Hoffert JD, Fenton RA, Moeller HB, Simons B, Tchapyjnikov D, McDill BW, Yu MJ, Pisitkun T, Chen F, Knepper MA: Vasopressin-stimulated increase in phosphorylation at Ser269 potentiates plasma membrane retention of aquaporin-2. *J Biol Chem* 283:24617-24627, 2008

5. Sandoval PC, Slentz DH, Pisitkun T, Saeed F, Hoffert JD, Knepper MA: Proteome-wide measurement of protein half-lives and translation rates in vasopressin-sensitive collecting duct cells. *J Am Soc Nephrol* 24:1793-1805, 2013

6. Tang F, Barbacioru C, Nordman E, Li B, Xu N, Bashkirov VI, Lao K, Surani MA: RNA-Seq analysis to capture the transcriptome landscape of a single cell. *Nat Protoc* 5:516-535, 2010

7. Lohse M, Bolger AM, Nagel A, Fernie AR, Lunn JE, Stitt M, Usadel B: RobiNA: a user-friendly, integrated software solution for RNA-Seq-based transcriptomics. *Nucleic Acids Res* 40:W622-W627, 2012

8. Dobin A, Davis CA, Schlesinger F, Drenkow J, Zaleski C, Jha S, Batut P, Chaisson M, Gingeras TR: STAR: ultrafast universal RNA-seq aligner. *Bioinformatics* 29:15-21, 2013

9. Heinz S, Benner C, Spann N, Bertolino E, Lin YC, Laslo P, Cheng JX, Murre C, Singh H, Glass CK: Simple combinations of lineage-determining transcription factors prime cis-regulatory elements required for macrophage and B cell identities. *Mol Cell* 38:576-589, 2010
